# Peak nasal inspiratory flow as outcome for provocation studies in allergen exposure chambers: a GA^2^LEN study

**DOI:** 10.1186/s13601-017-0169-4

**Published:** 2017-09-17

**Authors:** Georg Boelke, Uwe Berger, Karl-Christian Bergmann, Carsten Bindslev-Jensen, Jean Bousquet, Julia Gildemeister, Marek Jutel, Oliver Pfaar, Torsten Sehlinger, Torsten Zuberbier

**Affiliations:** 1 Charité – Universitätsmedizin Berlin, corporate member of Freie Universität Berlin, Humboldt-Universität zu Berlin, and Berlin Institute of Health, Department of Dermatology and Allergy, Allergy-Center-Charité, Berlin, Germany; 2 Department of Otorhinolaryngology, Aerobiology and Pollen Information Research Unit, Medical University of Vienna, Vienna, Austria; 3 Department of Dermatology and Allergy Centre, Odense University Hospital, Odense, Denmark; 4 CHRU, Montpellier University Hospital Center, Montpellier, France; 5 Mobile Chamber Experts GmbH, Berlin, Germany; 6 ALL-MED Medical Research Institute, Wrocław, Poland; 7 Department of Clinical Immunology, Wroclaw Medical University, Wrocław, Poland; 8 Department of Otorhinolaryngology, Head and Neck Surgery, Universitätsmedizin Mannheim, Medical Faculty Mannheim, Heidelberg University, Mannheim, Germany; 9 Center for Rhinology and Allergology, Wiesbaden, Germany; 10 Bluestone Technology GmbH, Woerrstadt, Germany

**Keywords:** Allergen exposure chamber (AEC), Allergy trial, GA^2^LEN chamber, Peak nasal inspiratory flow (PNIF), Provocation study

## Abstract

**Background:**

The GA^2^LEN chamber has been developed as a novel mobile allergen exposure chamber (AEC) allowing standardized multicenter trials in allergy. Hitherto, subjective nasal symptom scores have been the most often used outcome parameter, but in standardized modern trials objective parameters are preferred. Despite its practicability, the objective parameter peak nasal inspiratory flow (PNIF) has been rarely used for allergy trials in the setting of allergen exposure chambers. This study aims to evaluate PNIF as an outcome parameter for provocation studies in AECs.

**Methods:**

In a randomized controlled blinded setting subjects suffering from allergic rhinitis were exposed to grass pollen, birch pollen, house dust mite and/or placebo in the GA^2^LEN chamber. Different allergen concentrations were used to evaluate symptom severities. Patients had to perform PNIF before and every 30 min during a challenge using a portable PNIF meter.

**Results:**

86 subjects participated in 203 challenges, altogether. House dust mite provocations caused the greatest reduction in PNIF values, followed by grass pollen and birch pollen. Provocations with every allergen or pollen concentration led to a significant decrease (p < 0.05) in PNIF compared to baseline. Furthermore, positive correlations were obtained between PNIF and peak expiratory flow, height and weight, and inverse correlations between PNIF and total nasal symptom score, nasal congestion score and visual analog scale of overall subjective symptoms.

**Conclusion:**

PNIF is a helpful and feasible tool for conducting provocation trials with allergens, especially grass pollen and house dust mite, in an AEC.

**Electronic supplementary material:**

The online version of this article (doi:10.1186/s13601-017-0169-4) contains supplementary material, which is available to authorized users.

## Background

Depending on the geographic location and age of the patients, allergic rhinitis (AR) affects up to 10–40% of world’s population [1–3]. The prevalence of sensitization to airborne or indoor allergens reaches even higher values [4, 5]. Clinically AR presents especially with nasal congestion, sneezing, nasal pruritus and nasal discharge [6]. In Europe, major causes for seasonal allergic rhinitis are pollen from grass species (e.g. *Phleum pratense*), birch trees (betula) for northern Europe and olive (olea) for the Mediterranean regions, respectively, and house dust mites (HDM) and animal dander as the most common reason for perennial allergic rhinitis [7, 8]. AR is known to result in a decreased quality of life, sleep disorders, missing days at work or school, decreased productivity, and eventually causing direct and indirect medical costs of billions [9–11]. Hence, there is a great need for developing new treatment options and conducting clinical trials in the field of allergy. However, these trials are known to be time consuming due to their immanent demand on the pollen season. Furthermore, the amount of pollen each subject gets exposed to depends on several uncontrollable factors like climate, lifestyle and the actual pollen load in the air [12]. To overcome these difficulties, allergen exposure chambers (AEC) were developed and have been used for years in Europe, North America and Asia [13]. They provide a controlled, stable and reproducible environment regardless of the natural pollen season. Recently, the GA^2^LEN chamber was introduced, a mobile exposition chamber using a unique technique of exposure that allows individual allergen exposure for each patient during a challenge [14]. Besides subjective scoring through the patient itself during allergen challenges, there is a need for objective parameters as well. Out of the three most common methods to objectify nasal symptoms, namely rhinomanometry, acoustic rhinometry and peak nasal inspiratory flow (PNIF), the PNIF has been rarely used in the allergen chamber setting. It is cheap, portable and provides highly reproducible results despite depending on the patients’ cooperation [15]. Moreover, it correlates with subjective feeling of nasal obstruction, and is both easy and fast to learn [16–18]. PNIF measures the total nasal flow, therefore it is not dependent on the changing resistances between the left and right nostril during the nasal cycle. This study aims to evaluate PNIF as an outcome parameter for allergen provocations in an AEC. Moreover, associations between PNIF and biometric data (age, weight, height), PNIF and oral peak expiratory flow (PEF), and PNIF and subjective symptom scores and visual analog scales (VAS) are investigated.

## Methods

### Subjects

The study was conducted between January 2015 and May 2016 in Berlin, Germany. The majority of the trials were performed outside and only a few inside of the regional pollen season. Included were both male and female subjects between 18 and 75 years old with a history of AR caused by grass and/or birch pollen and/or house dust mite for at least two years, a positive skin prick test (SPT) (wheal diameter ≥3 mm than negative control) for grass mix, birch and/or house dust mite (*Dermatophagoides pteronyssinus* and/or *Dermatophagoides farinae*), and/or ImmunoCAP score ≥2 for the allergen they were exposed to. Both smokers and non-smokers were included in this study. Exclusion criteria were pregnancy, acute or chronic rhinosinusitis, severe asthma, prior immunotherapy, and treatment with a nasal decongestant, nasal glucocorticoid, oral antihistamine, oral chromone derivates (up to 7 days prior to exposure) or systemic glucocorticoids (up to 30 days prior to exposure). Every patient gave written informed consent prior to exposure. The study was approved by the ethical committee of the Charité (No. EA1/193/14 for grass/birch and EA1/152/15 for HDM) and conducted following the guidelines from the Declaration of Helsinki.

### Methods and material

Subjects were included based on history and skin test independent of symptom severity during an exposure. The study was planned as modified double-blinded and placebo-controlled, patients did neither know if they were exposed to an allergen nor the amount of the allergen. Right before each provocation, patients were randomly assigned to a seat using a randomization software. Beforehand, particle disperse units above each seat had been prepared by a technician uninvolved in the interactions between investigators and patients. The investigators were sitting in a separated control room exposing the patients according to a preset randomized pattern only revealed at the start without any interactions during the exposure with the patients to ensure a completely blinded study. Patients were exposed for 90–240 min for grass/birch pollen and 60–90 min for HDM. To get comparable results, we focus on data from exposures for at least 120 min regarding grass/birch pollen and 90 min for HDM. Before the challenge began, patients had to sit on their designated seats for at least 15 min to get acclimated. Before and every 10 min during the challenge, patients had to evaluate their nasal symptoms (itching, sneezing, rhinorrhea, congested nose) on a symptom check card using a rating scale ranging from zero points (no symptoms present) to three points (severest symptoms present). All four symptoms were summed up to the total nasal symptom score (TNSS) with a highest possible score of 12 points. Moreover, before exposure started and every 30 min during exposure patients measured their PEF using a portable peak flow meter (PFM20, Omron Healthcare Europe, Hoofddorp, Netherlands) and their PNIF using a portable PNIF meter (In-check, Inspiratory flow meter, Clement Clarke International, Essex, UK). Before the baseline measurements were conducted, each patient was given a short training about how to perform the test correctly and to have some trials to avoid a training effect. Each measurement was taken in a seated position, the best of at least two successful measurements was noted. PNIF und PEF meter were kept in a closable bag next to the patient all throughout the exposure to protect them from contamination with allergen and were only taken out for the measurements. The patients evaluated their overall subjective symptoms being asked to assess their present general well-being using a 10 cm (cm) visual analog scale (VAS) ranging from very good (0 cm) to very bad (10 cm) directly before and every 30 min after the start of each challenge. Immediately before and after each challenge participants underwent spirometry for safety. Patients could participate multiple times and if eligible for each allergen and their different concentrations. For this study, only their last visit for each allergen and its different concentrations was included to avoid duplicates.

### Allergen exposure chamber

The GA^2^LEN chamber consists of two standard 24 ft. containers (one for observation and storage, the other one for the exposition chamber itself), the outer dimensions are 7.43 × 5.10 × 2.86 m (length × width × height). It can contain up to nine patients per run. Each patient gets their individual allergen exposure. Due to strong laminar airflow on both sides of the exposition chamber and almost no airflow in the area where the subjects are seated, there is no mixture of air and the contained allergen between the subjects guaranteeing their individual exposure. Detailed information on the technical aspect has been published by Zuberbier et al. [14]. Climate conditions were permanently controlled all throughout the exposures [temperature was set at 20.5 °C (±0.5 K), humidity at 55% (±5%)]. Patients were exposed to 4000 and 8000 grains/m^3^ *Phleum pratense*, 4000, 8000 and 16,000 grains/m^3^ *Betula pendula* (both *Allergon AB*, *Ängelholm*, *Sweden*), and 250 µg/m^3^ house dust mite raw material (computed value, consists of whole bodies, body parts and feces; GMP material, equivalent to 400 ng Der p 1/m^3^).

### Statistics

Data was analyzed and diagrams were created with the help of IBM SPSS Statistics Version 24.0 for Windows (Armonk, NY: IBM Corp.) and Microsoft Excel 2013 (Redmond, WA). The challenges were initially performed as validation trials for the chamber, thus no specific power analysis for differences in PNIF was calculated. To compare between the groups, the relative PNIF value in percent was computed (PNIF%). Therefore, the subject’s baseline PNIF was determined as 100%. PNIF% is reported as medians with bias corrected and accelerated bootstrap 95% confidence intervals of the median (95% BCa CI), absolute PNIF values as mean ± standard deviation (SD). A p level of <0.05 was accepted as significant. Kruskal–Wallis test and Mann–Whitney-U test were used for comparisons between the different treatment groups, Friedman test was used when differences in-between a group were examined. Pairwise comparisons as post hoc tests were computed using the Dunn–Bonferroni approach. Spearman rank correlations were calculated to assess associations between PNIF and age, height, weight, PEF, nasal congestion score, TNSS and VAS. For correlations with baseline PNIF, each subject was only included once with their best PNIF baseline value. For correlations between PNIF and subjective symptom scores and VAS, each challenge was included using its mean PNIF%, its mean TNSS and nasal congestion score from beginning to end of exposure, and its mean VAS during a provocation test minus baseline VAS, respectively. Area under the curve (AUC) was calculated using the trapezoid rule and is reported as medians and 95% BCa CI.

## Results

86 patients were included, 47 of them were female (54.7%). Men had a mean PNIF at baseline of 174.2 (±SD 59.9 L/min) and women 126.3 (±SD 31.0 L/min). No differences were found for PNIF at baseline between in- and out-side the pollen season provocations in the chamber. Detailed demographics are described in Table 1.

**Table 1:** Patient demographics

Parameter (n = 86)	Male, n = 39 (45.3%)	Female, n = 47 (54.7%)
Age in years, mean (range)	29.3 (19–74)	26.4 (19–47)
Height in m, mean (range)	1.83 (1.72–1.96)	1.69 (1.55–1.80)
Weight in kg, mean (range)	78.5 (54–96)	62.9 (47–88)
Active smokers (%)	6 (15.4%)	4 (8.5%)
Sensitization to grass (%)	32 (82.1%)	39 (83%)
Sensitization to birch (%)	29 (74.4%)	38 (80.9%)
Sensitization to house dust mite (%)	23 (59%)	32 (68.1%)
PNIF in L/min, mean (± SD)	174.2 (±59.9)	126.3 (±31.0)
PEF in L/min, mean (± SD)	588.3 (±83.8)	401.3 (±75.0)
FEV1% predicted, mean (± SD)	92.2 (±11.5)	89.6 (±12.0)

*FEV1* forced expiratory volume in one second, *PNIF* peak nasal inspiratory flow, *PEF* peak expiratory flow, *SD* standard deviation

### Grass

**Fig. 1:**
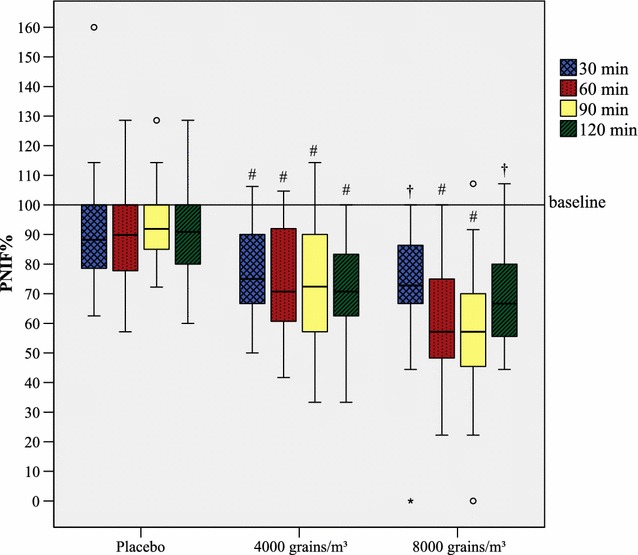
Reduction of PNIF during exposure with grass pollen in the GA^2^LEN chamber. PNIF development during exposure with *Phleum pratense*. A hash marks a reduction compared to baseline p < 0.001, a dagger a reduction compared to baseline p < 0.01. Outliers are presented as degree sign, extreme outliers as asterisk. PNIF% from both actively exposed groups (4000 and 8000 grains/m^3^) is significantly lower (p < 0.05) than in the placebo group at every associated time of measurement. PNIF% is displayed as medians and boxplots

34 subjects were tested with 4000 grains/m^3^ of grass pollen, 22 subjects with 8000 grains/m^3^ and 22 subjects with placebo. Mean reduction from baseline PNIF was 32.4 (±SD 20.9 L/min) in the 4000 grains/m^3^ group, 45.3 (±SD 23.7 L/min) in the 8000 grains/m^3^ group and 12.0 (±SD 14.9 L/min) in the placebo group (Table 2). The relative PNIF compared to baseline (PNIF%) reduction was 29.7%, 95% CI (20.1, 32.8) for 4000 grains/m^3^, 36.8%, 95% CI (27.8, 43.8) for 8000 grains/m^3^ and 8.9%, 95% CI (1.3, 15.7) for placebo (Additional file 1: Table S1). Kruskal–Wallis-test found significant differences between the PNIF% values of the groups after 30 min (χ^2^ = 10.357, p = 0.006), after 60 min (χ^2^ = 22.390, p < 0.001), after 90 min (χ^2^ = 26.829, p < 0.001) and after 120 min (χ^2^ = 20.789, p < 0.001). Post-hoc tests revealed significant differences for both 4000 and 8000 grains/m^3^ compared to placebo at each time of measurement (Fig. 1). Furthermore, a significant difference between both active groups could be detected for PNIF% values at 60 min (z = 15.004, p = 0.046). Friedman test showed significant differences in each of the both active groups for every PNIF% value compared to their baseline, whereas no significant difference could be computed in the placebo group at all. The AUC for PNIF% was significant lower for both active groups [4000 grains/m^3^ 8957.6 (8433.0, 10,046.6); 8000 grains/m^3^ 8241.7 (7278.1, 9055.6)] compared to placebo [11,114.6 (10,331.8, 11,766.7)] (p < 0.001).

**Table 2:** PNIF values (in L/min) for challenges with grass pollen, birch pollen and house dust mite (HDM)

Pollen	Concentration	PNIF baseline (±SD)	PNIF 30 min (±SD)	PNIF 60 min (±SD)	PNIF 90 min (±SD)	PNIF 120 min (±SD)
Grass	Placebo	123.4 (±55.4)	106.8 (±40.4)	109.3 (±44.9)	114.3 (±51.1)	115.2 (±56.3)
	4000 grains/m^3^	130.7 (±54.8)	102.7 (±50.9)	99.3 (±52.7)	95.7 (±52.5)	95.6 (±50.7)
	8000 grains/m^3^	135.5 (±52.7)	98.0 (±50.9)	84.1 (±46.3)	82.5 (±47.6)	96.1 (±48.3)
Birch	Placebo	132.5 (±63.1)	118.6 (±58.8)	116.4 (±56.9)	112.7 (±51.2)	117.5 (±56.9)
	4000 grains/m^3^	143.6 (±37.5)	126.1 (±42.4)	118.9 (±41.7)	117.9 (±37.2)	120.2 (±44.2)
	8000 grains/m^3^	140.8 (±43.3)	122.7 (±44.7)	115.6 (±45.6)	119.9 (±40.5)	118.9 (±39.5)
	16,000 grains/m^3^	143.6 (±47.2)	118.6 (±47.8)	110.0 (±43.6)	108.6 (±53.5)	114.6 (±50.3)
HDM	Placebo	136.9 (±46.4)	121.9 (±50.1)	106.1 (±52.0)	104.4 (±51.3)	X
	250 µg/m^3^	139.4 (±55.1)	95.2 (±38.6)	80.0 (±38.0)	81.5 (±40.2)	X

*PNIF* peak nasal inspiratory flow, *SD* standard deviation

### Birch

**Fig. 2:**
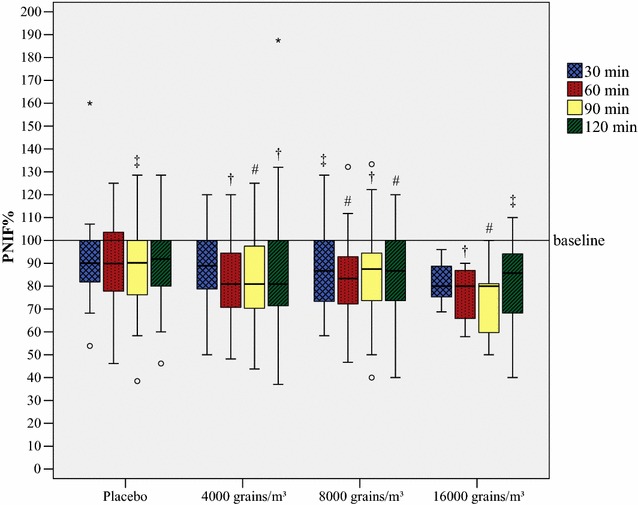
Reduction of PNIF during exposure with birch pollen in the GA^2^LEN chamber. PNIF development during exposure with *Betula pendula*. A hash marks a reduction compared to baseline p < 0.001, a dagger a reduction compared to baseline p < 0.01 and a double dagger a reduction compared to baseline p < 0.05. Outliers are presented as degree sign, extreme outliers as asterisk. PNIF% is displayed as medians and boxplots

28 subjects were challenged with 4000 grains/m^3^ of birch pollen, 33 subjects with 8000 grains/m^3^, 11 subjects with 16,000 grains/m^3^ and 22 subjects with placebo. PNIF dropped from baseline during exposure 22.8 (±SD 25.3 L/min) for 4000 grains/m^3^ concentration [PNIF% reduction 15.4%, 95% CI (8.8, 20.5)], 21.5 (±SD 23.6 L/min) for 8000 grains/m^3^ [12.0%, 95% CI (9.4, 21.7)], 30.7 (±SD 21.9 L/min) for 16,000 grains/m^3^ [19.6%, 95% CI (12.5, 28.4)], and 16.2 (±SD 22.6 L/min) for placebo [8.5%, (1.3, 17.1)] (Table 2; Additional file 1: Table S1). Friedman test found significant differences for every actively exposed group compared to their baseline value in PNIF%. In detail, at challenges with 8000 grains/m^3^ each point of measurement differed significantly from the baseline, whereas at challenges with 4000 and 16,000 grains/m^3^ each point of measurement from minute 60 and further on did. In the placebo group, only at point of measurement at minute 90 a significant difference compared to baseline could be found (Fig. 2). However, PNIF% showed no significant difference between the challenge groups, even though a trend was clearly recognizable. Hence, the three groups that got actively exposed to birch pollen were summarized into one active group. In addition, only those tests runs were included where test subjects reached a TNSS greater than two points on at least two symptom check cards. Eventually, 38 challenges were included into the active group, the placebo group remained the same. Mean reduction from baseline for absolute PNIF values in the active group was 31.2 (±SD 24.8 L/min) and 20.4%, 95% CI (15.8, 25.0) for relative values (Additional files 2, 3: Tables S2, S3). Values in the placebo group stayed the same as reported earlier. Mann–Whitney-U test found significant differences when comparing the PNIF% values between active and placebo group after 60 min (z = −2.809, p = 0.005), after 90 min (z = −2.380, p = 0.017) and after 120 min (z = −2.133, p = 0.033) (Additional file 4: Fig. S1). Moreover, the AUC of PNIF% was significantly lower in the active group [9878.6 (9115.4, 10,250.0)] than in the placebo group [11,092.9 (10,105.3, 11,921.1)] (z = −2.754, p = 0.006).

### House dust mite

**Fig. 3:**
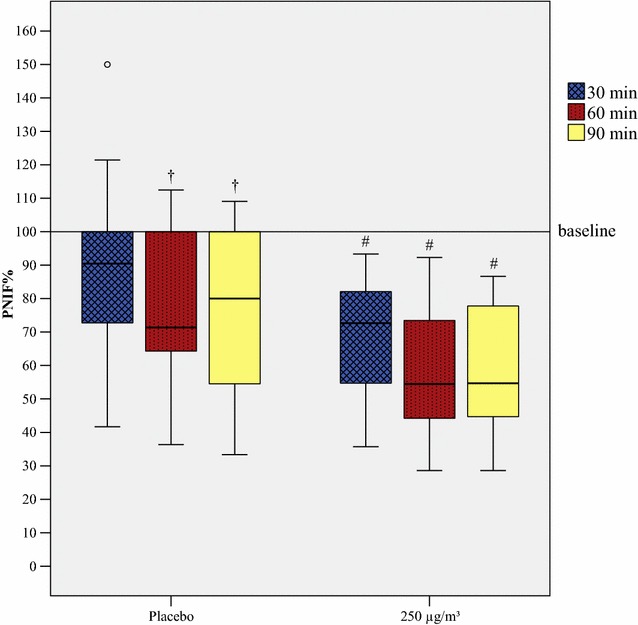
Reduction of PNIF during exposure with house dust mite (Der p 1) in the GA^2^LEN chamber. PNIF development during exposure with house dust mite material. A hash marks a reduction compared to baseline p < 0.001, a dagger a reduction compared to baseline p < 0.01. Outliers are presented as degree sign. PNIF% from the actively exposed group (250 µg/m^3^) is significantly lower (p < 0.05) than in the placebo group at every associated time of measurement. PNIF% is displayed as medians and boxplots

24 patients were exposed to 250 µg/m^3^ HDM material, 18 patients participated in a placebo run. Mean change from baseline was 53.8 (±SD 33.9 L/min) in the active group and 26.1 (±SD 28.7 L/min) in the placebo group for absolute values (Table 2; Additional file 5: Fig. S2), 40.1%, 95% CI (25.8, 44.4) and 20.7%, 95% CI (6.1, 33.3) for relative values, respectively (Additional file 1: Table S1). Mann–Whitney-U test found significant differences between both groups regarding their PNIF% values after 30 min (z = −2.975, p = 0.003), after 60 min (z = −2.328, p = 0.020) and after 90 min (z = −2.327, p = 0.020) (Fig. 3). Similar to the other conducted challenges with grass and birch, comparisons of absolute PNIF values found no significant difference due to the unequal baselines. AUC for PNIF% was significant lower in the active group [6156.4 (5666.7, 7100.0)] than in the placebo group [7440.0 (6458.8, 8727.3)] (z = −2.872, p = 0.004). Furthermore, Friedman test showed a significant difference at each point of measurement during exposure compared to baseline in the active group. Though, the placebo group differed also significantly from their baseline value at points of measurement after 60 min and after 90 min.

### Comparison between the allergens

Please find these results in the Additional file 6: Appendix S1 and Additional file 7: Figure S3.

### Correlations

**Fig. 4:**
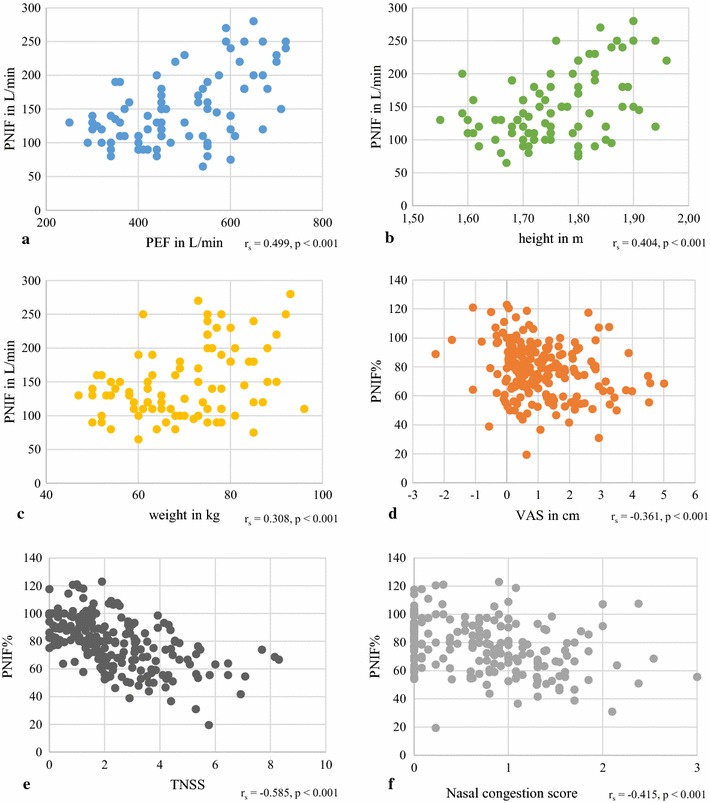
Correlations between peak nasal inspiratory flow (PNIF) and oral peak expiratory flow (PEF) (**a**), height (**b**), and weight (**c**) with n = 86, and relative peak nasal inspiratory flow compared to baseline (PNIF%) and mean VAS change from baseline (**d**), mean Total Nasal Symptom Score (TNSS) (**e**), and mean nasal congestion score (**f**) with n = 203

Positive weak to moderate correlations could be found between PNIF and PEF (r_s_ = .499, p < 0.001), PNIF and height (r_s_ = .404, p < 0.001) and PNIF and weight (r_s_ = .308, p < 0.001). A correlation between PNIF and age was not visible (r_s_ = .005, p = 0.96). Furthermore, an inverse moderate to strong correlation could be computed between PNIF% and TNSS (r_s_ = −.585, p < 0.001), as well as inverse weak to moderate correlations between PNIF% and nasal congestion score (r_s_ = −.415, p < 0.001), and PNIF% and VAS of overall subjective symptoms (r_s_ = −.361, p < 0.001) (Fig. 4).

## Discussion

PNIF has been recommended and been used as an outcome parameter in allergen immunotherapy trials [19, 20], pharmacological trials [21, 22], nasal allergen challenges [23, 24], before surgical interventions [25, 26], and is also a feasible tool in assessing nasal patency in both children [27] and adults [28, 29]. This was the first study to evaluate peak nasal inspiratory flow as an outcome parameter in an allergen exposure chamber. Overall, 86 subjects participated in 203 individual challenges with either grass pollen, birch pollen, house dust mite material or placebo. At baseline, male subjects reached a PNIF of 174.2 ± 59.9 L/min and female subjects of 126.3 ± 31.0 L/min. Measurements were taken in a seated position, as there exists no significant difference to standing position [30], and the best of at least two successful measurements was noted due to no additional benefit in a third trial [15]. Reproducibility and no demand for priming exposures were previously reported [14]. Our results confirm a study by Denguezli Bouzgarou et al. who found almost exact same values in a healthy North African population with a mean PNIF in male subjects of 174 ± 54 and 126 ± 33 L/min in female subjects [31]. Looking at data for a European population our values were lower than data obtained by Åkerlund et al. [32], but comparable to findings from Ottaviano et al. with a PNIF of 143 ± 48.6 L/min for male and 121.9 ± 36 L/min for female [33]. A study by Klossek et al. in a French population found clearly lower normal ranges in PNIF though. Even when only reporting the values obtained from the subjects, who reported no nasal discomfort at all, men had a mean PNIF of 100.3 ± 43.6 L/min and women of 79.3 ± 32.2 L/min [34]. However, an explanation for these low values was not found. The greatest reduction in PNIF was elicited by HDM in our study, followed by grass pollen and birch pollen. PNIF also decreased mildly in the placebo group, even when no patient in the chamber was exposed to an allergen. Whether the decline results apart from the placebo effect itself, from decreasing patients’ effort during the exposure, increased osmolarity of nasal mucus due to increased ventilation from the measurements, or despite 55% humidity too dry air, needs to be further investigated. Standard deviation of some results for absolute PNIF values exceeded the mean value caused by the unequal distribution. Hence, it is of utmost importance to compare the relative reductions. Decreased PNIF is known in HDM allergy as allergic subjects usually present with nasal obstruction [35]. However, little is known about the differences in nasal symptoms elicited by different airborne pollen. In our challenges PNIF decreased in subjects exposed to grass pollen much greater in both absolute and relative values than in subjects exposed to birch pollen. Nonetheless, both kinds of pollen had in common that the more the pollen concentration increased the more PNIF reduction was induced. These results imitate the conditions in nature as described by Caillaud et al. who described a linear relationship between birch pollen concentration and symptoms elicited until symptom severity reaches a plateau when a certain threshold concentration is exceeded [36]. As demanded by a recently published position paper from the European Academy of Allergy and Clinical Immunology (EAACI) it is important to compare the obtained results between the existing exposure chambers [37]. To the authors knowledge only two studies conducted in an Environmental Exposure Unit (EEU) in Kingston, Ontario have used PNIF as an outcome parameter in clinical trials [38, 39]. Both studies were clinical evaluations of the EEU for birch pollen and grass pollen exposure, respectively. Focusing just on the reported PNIF data for provocations with grass pollen, the mean reduction of PNIF after 180 min of exposure compared to baseline to either 2500 or 3500 grains/m^3^ grass pollen (*Lolium perenne*) was 29.8 and 42.9 L/min, respectively, resulting in a relative reduction of 30.4 and 34.2%, respectively. These results match our findings with a PNIF reduction of 35.2 L/min (relative reduction 29.3%) after 120 min exposure to 4000 grains/m^3^ of grass pollen compared to baseline. However, allergic patients were not provoked to placebo in the EEU, thus the effect of the chamber itself to allergic subjects is unknown. Furthermore, the technology of pollen distribution is totally different in both chambers. Whereas in the EEU and most of the other existing chambers pollen gets distributed via fans all over the exposition room, the GA^2^LEN chamber provides an individual exposure to every subject giving an exact knowledge of the concentration every test subject got exposed to. Hence, even when using the same allergen concentration the results might not be directly comparable. Both chambers provoked less reduction in PNIF during challenges with birch pollen. That is why it can be suspected that birch allergy elicit less nasal congestion and other symptoms are more present. This needs to be further evaluated. In our study, we found moderate positive correlations between PNIF and weight, height and oral peak inspiratory flow. Even though some publications denied a correlation between PNIF and weight [32] or PNIF and height [40], other studies confirmed these associations, especially for PNIF and PEF [41–43]. In our study PNIF and subjective nasal symptoms were found to correlate inversely with a Spearman’s rank correlation coefficient r_s_ = −0.59 between PNIF and TNSS, and r_s_ = −0.42 between PNIF and nasal congestion score. Other studies, that were using exactly the same TNSS as we did, computed correlations from −0.50 to −0.62 between PNIF and TNSS, confirming our analysis and thus consolidate the usefulness of PNIF as an objective control parameter for subjective symptoms [44, 45]. Furthermore, the publication from Ellis et al. reported a weak to moderate negative correlation from −0.32 to −0.37 between PNIF and subjective scoring of nasal congestion, which can be validated and even enhanced with data obtained in the GA^2^LEN chamber [38]. The correlation between PNIF and VAS of overall subjective symptoms was found to be at −0.36 in the GA^2^LEN chamber, thus being in the range of already published correlations of −0.39 to −0.48 between PNIF and VAS [17, 28, 29]. However, these studies focused only on the VAS of nasal obstruction in particular. Hence, our findings provide additional information about the relation of PNIF and the actual patient’s perception of their overall symptom severity, which possibly represents real-life conditions more accurately.

## Conclusions

In conclusion, due to its portability, simple application and good correlation to subjective symptoms, PNIF is a valuable tool for provocation trials in AECs. However, more clinical trials comparing this outcome in different AECs facilities would be advisable.

## Additional files

**Additional file 1: Table S1.** PNIF% values for challenges with grass pollen, birch pollen and house dust mite (HDM).

**Additional file 2: Table S2.** PNIF values for birch challenges (in L/min). Patients in active group were only included when they experienced a Total Nasal Symptom Score (TNSS) greater than 2 points on at least two symptom check cards.

**Additional file 3: Table S3.** PNIF% values for birch challenges. Patients in active group were only included when they experienced a Total Nasal Symptom Score (TNSS) greater than 2 points on at least two symptom check cards.  

**Additional file 4: Figure S1.** Reduction of PNIF during exposure with birch pollen in the GA²LEN chamber. PNIF development during exposure with *Betula pendula*. Every challenge to birch pollen got pooled into one active group and only those runs were included where a TNSS greater than 2 points was reported on at least two symptom check cards throughout the whole challenge. A hash marks a reduction compared to baseline p < 0.001, a dagger a reduction compared to baseline p < 0.01, a double dagger a reduction compared to baseline p < 0.05. Outliers are presented as degree sign, extreme outliers as asterisk. PNIF% in the active group differed significantly (p < 0.05) from the placebo group at 60, 90 and 120 min. PNIF% is displayed as medians and boxplots.

**Additional file 5: Figure S2.** Example of individual PNIF development (in L/min) for every subject when exposed to house dust mite (**a** placebo, **b** 250 µg/m³).

**Additional file 6: Appendix S1.** Comparison between the different allergens.

**Additional file 7: Figure S3.** Comparison of different allergens and their PNIF outcome. PNIF development compared between the different allergens and placebo. Both grass pollen and house dust mite (HDM) elicited significantly greater PNIF% reductions at each associated time of measurement than placebo (p < 0.001) and birch pollen (p < 0.01). PNIF% is displayed as medians and boxplots. Outliers are presented as degree sign, extreme outliers as asterisk.

## Abbreviations

AEC: allergen exposure chamber
AR: allergic rhinitis
AUC: area under the curve
cm: centimeter
FEV1: forced expiratory volume in one second
GA^2^LEN: Global Asthma and Allergy European Network
HDM: house dust mite
min: minutes
PEF: oral peak expiratory flow
PNIF: peak nasal inspiratory flow
SD: standard deviation
SPT: skin prick test
TNSS: total nasal symptom score
VAS: visual analog scale
95% BCa CI: bias corrected and accelerated bootstrap 95% confidence interval of the median
