# Supplementary material for: Peak nasal inspiratory flow as outcome for provocation studies in allergen exposure chambers: a GA2LEN study
Source: Clin Transl Allergy. 2017 Sep 17;7:33. doi: 10.1186/s13601-017-0169-4 (PMC5604509; doi:10.1186/s13601-017-0169-4)
Supplement: Supplementary file 1 — Additional file 1: Table S1. PNIF% values for challenges with grass pollen, birch pollen and house dust mite (HDM). [file 13601_2017_169_MOESM1_ESM.docx]

**Table S1** - PNIF% values for challenges with grass pollen, birch pollen and house dust mite (HDM)

| **Pollen** | **Concentration** | **PNIF% 30 min**  **[95% BCa CI]** | **PNIF% 60 min**  **[95% BCa CI]** | **PNIF% 90 min**  **[95% BCa CI]** | **PNIF% 120 min**  **[95% BCa CI]** |
| --- | --- | --- | --- | --- | --- |
| **Grass** | placebo | 88.2 [79.6, 100.0] | 89.9 [82.3, 100.0] | 91.9 [86.7, 100.0] | 90.9 [87.5, 100.0] |
|  | 4000 grains/m³ | 75.0 [71.4, 82.3] | 70.7 [63.6, 84.6] | 72.4 [60.4, 83.3] | 70.7 [65.7, 76.5] |
|  | 8000 grains/m³ | 72.8 [66.7, 83.3] | 57.2 [50.0, 66.7] | 57.2 [51.3, 66.7] | 66.7 [60.4, 74.5] |
| **Birch** | Placebo | 90.0 [87.5, 100.0] | 89.9 [78.9, 100.0] | 90.2 [76.7, 100.0] | 91.9 [84.4, 95.2] |
|  | 4000 grains/m³ | 88.9 [81.8, 92.9] | 80.9 [72.7, 93.3] | 80.9 [75.0, 90.9] | 80.9 [72.4, 93.0] |
|  | 8000 grains/m³ | 86.7 [78.9, 91.7] | 83.3 [76.9, 88.0] | 87.5 [83.3, 91.7] | 86.7 [82.1, 93.1] |
|  | 16000 grains/m³ | 80.0 [74.4, 89.3] | 80.0 [78.6, 80.0] | 80.0 [68.9, 80.0] | 85.7 [73.1, 92.3] |
| **HDM** | Placebo | 90.5 [78.6, 100.0] | 71.4 [64.3, 97.4] | 80.0 [75.0, 89.4] | X |
|  | 250 µg/m³ | 72.7 [59.3, 81.8] | 54.4 [47.1, 68.3] | 54.7 [48.1, 73.3] | X |

PNIF – peak nasal inspiratory flow; 95% BCa CI – Bias corrected and accelerated 95% confidence interval of the median
