# Supplementary material for: Peak nasal inspiratory flow as outcome for provocation studies in allergen exposure chambers: a GA2LEN study
Source: Clin Transl Allergy. 2017 Sep 17;7:33. doi: 10.1186/s13601-017-0169-4 (PMC5604509; doi:10.1186/s13601-017-0169-4)
Supplement: Supplementary file 2 — Additional file 2: Table S2. PNIF values for birch challenges (in L/min). Patients in active group were only included when they experienced a Total Nasal Symptom Score (TNSS) greater than 2 points on at least two symptom check cards. [file 13601_2017_169_MOESM2_ESM.docx]

**Table S2** - PNIF values for birch challenges (in L/min). Patients in active group were only included when they experienced a Total Nasal Symptom Score (TNSS) greater than 2 points on at least two symptom check cards.

| **Group** | **PNIF baseline**  **(± SD)** | **PNIF 30 min**  **(± SD)** | **PNIF 60 min**  **(± SD)** | **PNIF 90 min**  **(± SD)** | **PNIF 120 min**  **(± SD)** |
| --- | --- | --- | --- | --- | --- |
| Placebo | 132.5 (± 63.1) | 118.6 (± 58.8) | 116.4 (± 56.9) | 112.7 (± 51.2) | 117.5 (± 56.9) |
| Active | 144.6 (± 42.9) | 121.2 (± 44.7) | 109.7 (± 45.0) | 111.6 (± 38.4) | 111.3 (± 43.1) |

PNIF – peak nasal inspiratory flow; SD – standard deviation
