# Supplementary material for: Peak nasal inspiratory flow as outcome for provocation studies in allergen exposure chambers: a GA2LEN study
Source: Clin Transl Allergy. 2017 Sep 17;7:33. doi: 10.1186/s13601-017-0169-4 (PMC5604509; doi:10.1186/s13601-017-0169-4)
Supplement: Supplementary file 3 — Additional file 3: Table S3. PNIF% values for birch challenges. Patients in active group were only included when they experienced a Total Nasal Symptom Score (TNSS) greater than 2 points on at least two symptom check cards. [file 13601_2017_169_MOESM3_ESM.docx]

**Table S3** - PNIF% values for birch challenges. Patients in active group were only included when they experienced a Total Nasal Symptom Score (TNSS) greater than 2 points on at least two symptom check cards.

| **Group** | **PNIF% 30 min**  **[95% BCa CI]** | **PNIF% 60 min**  **[95% BCa CI]** | **PNIF% 90 min**  **[95% BCa CI]** | **PNIF% 120 min**  **[95% BCa CI]** |
| --- | --- | --- | --- | --- |
| Placebo | 90.0 [87.5, 100.0] | 89.9 [78.9, 100.0] | 90.2 [76.7, 100.0] | 91.9 [84.4, 95.2] |
| Active | 81.5 [77.4, 90.9] | 72.7 [70.0, 79.4] | 80.0 [75.0, 81.3] | 77.0 [66.7, 87.0] |

PNIF – peak nasal inspiratory flow; 95% BCa CI – Bias corrected and accelerated 95% confidence interval of the median
