# Supplementary figures and images for: Peak nasal inspiratory flow as outcome for provocation studies in allergen exposure chambers: a GA2LEN study
Source: Clin Transl Allergy. 2017 Sep 17;7:33. doi: 10.1186/s13601-017-0169-4 (PMC5604509; doi:10.1186/s13601-017-0169-4)

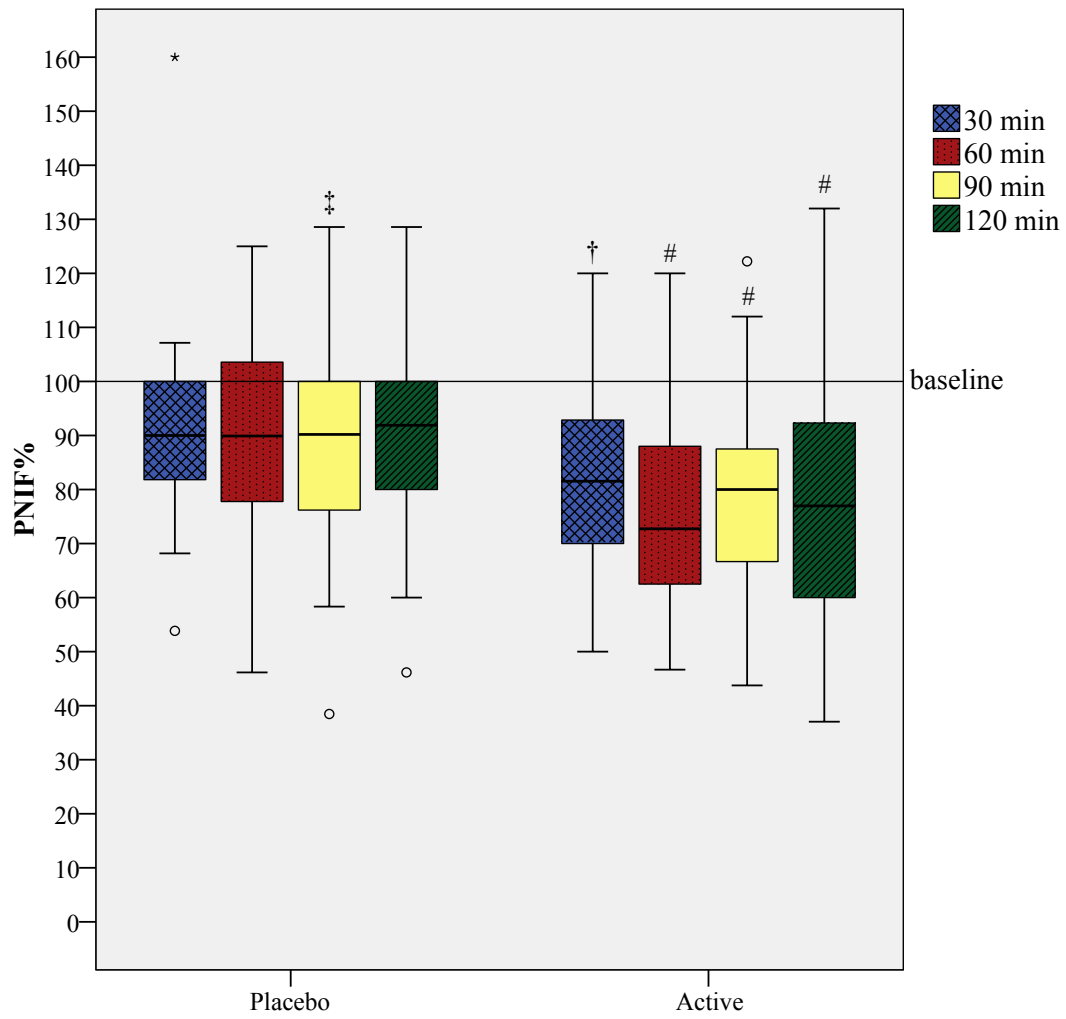

Supplement: Supplementary file 4 — Additional file 4: Figure S1. Reduction of PNIF during exposure with birch pollen in the GA²LEN chamber. PNIF development during exposure with Betula pendula. Every challenge to birch pollen got pooled into one active group and only those runs were included where a TNSS greater than 2 points was reported on at least two symptom check cards throughout the whole challenge. A hash marks a reduction compared to baseline p < 0.001, a dagger a reduction compared to baseline p < 0.01, a double dagger a reduction compared to baseline p < 0.05. Outliers are presented as degree sign, extreme outliers as asterisk. PNIF% in the active group differed significantly (p < 0.05) from the placebo group at 60, 90 and 120 min. PNIF% is displayed as medians and boxplots. [file 13601_2017_169_MOESM4_ESM.pdf]

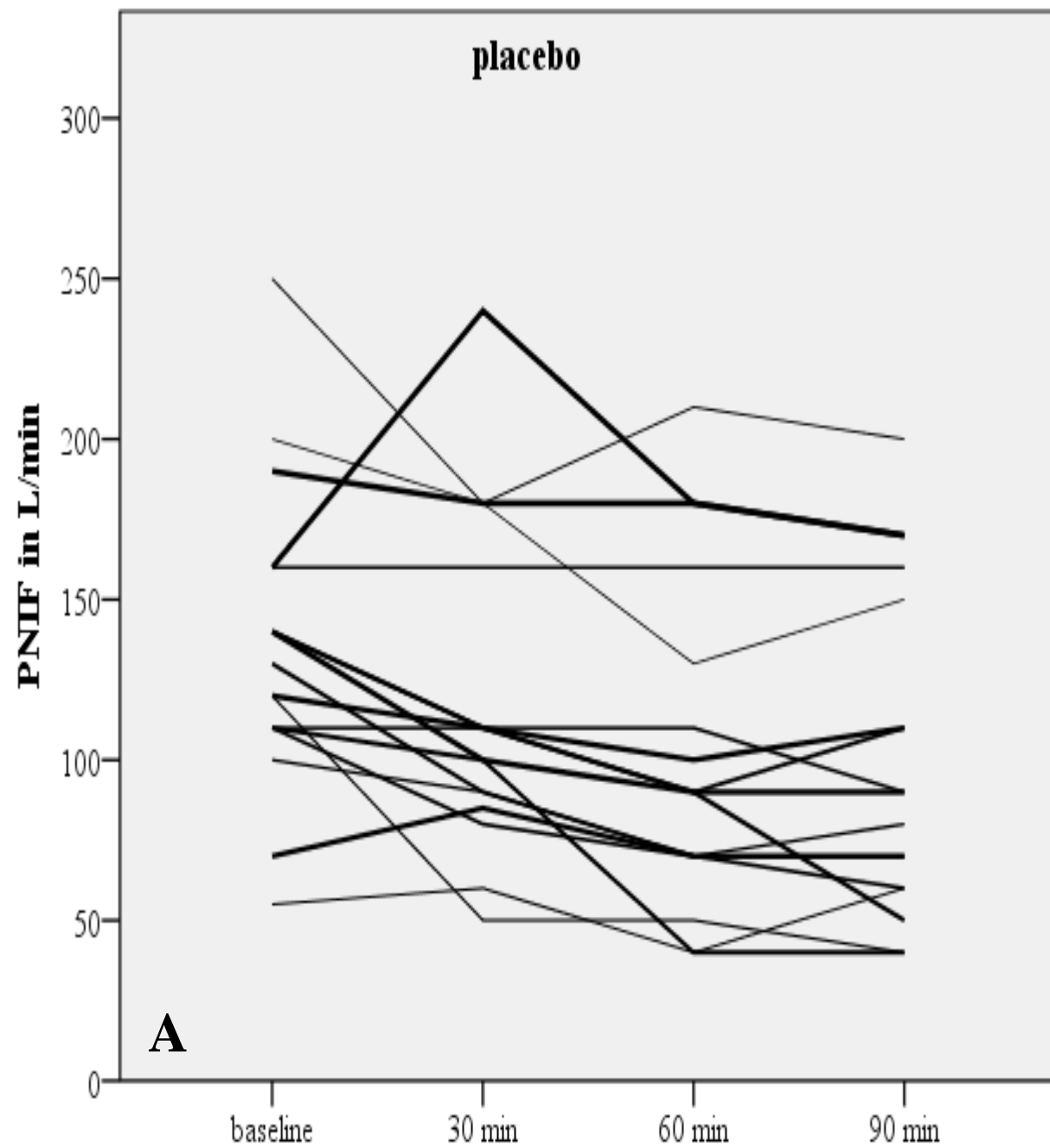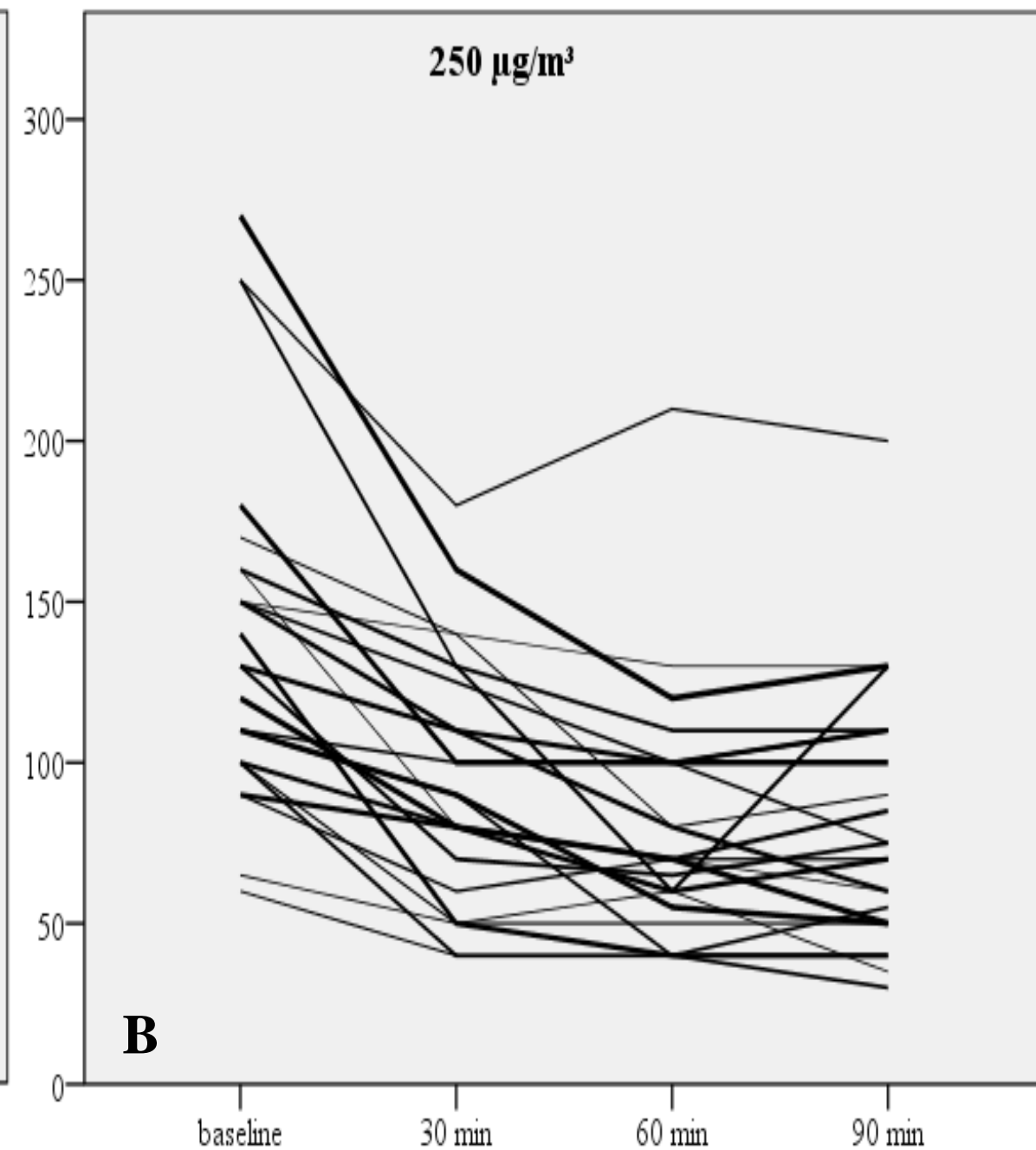

Supplement: Supplementary file 5 — Additional file 5: Figure S2. Example of individual PNIF development (in L/min) for every subject when exposed to house dust mite (a placebo, b 250 µg/m³). [file 13601_2017_169_MOESM5_ESM.pdf]

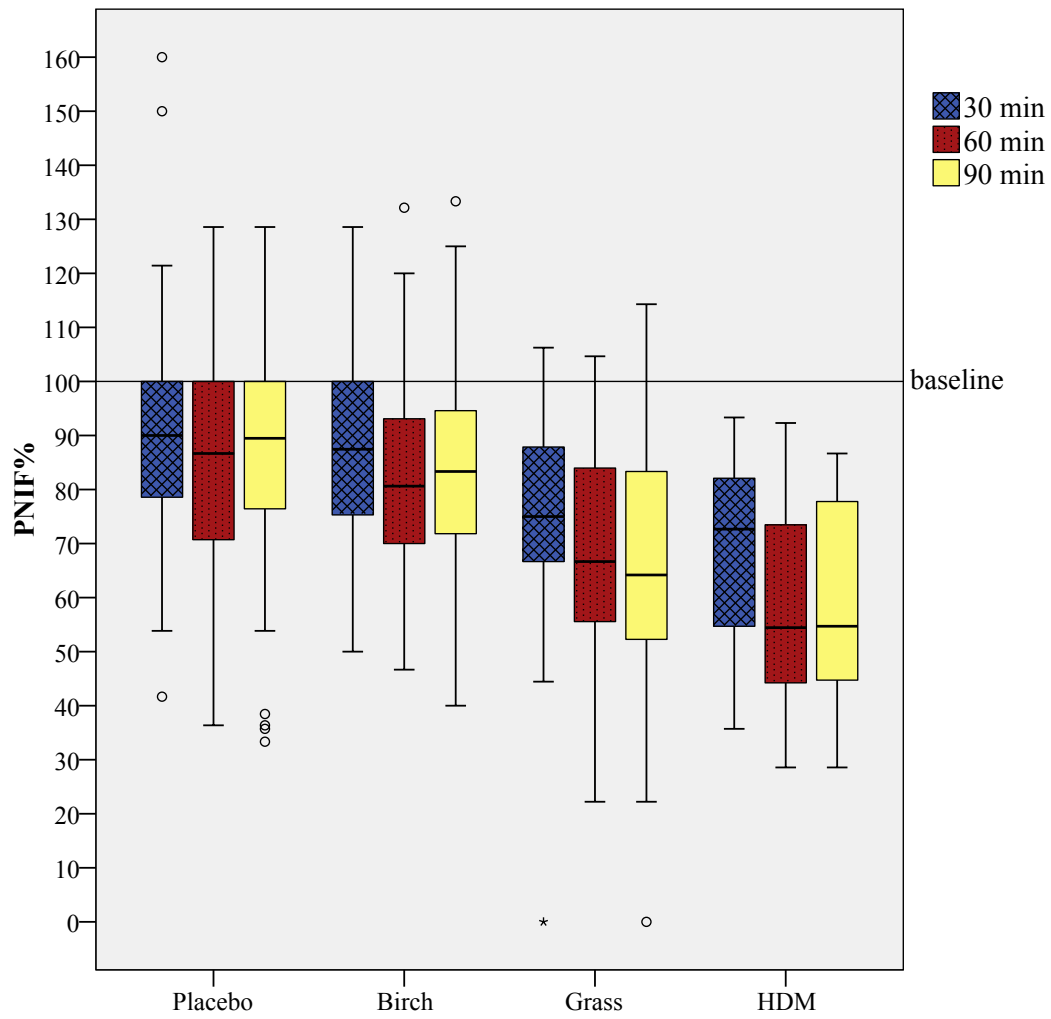

Supplement: Supplementary file 7 — Additional file 7: Figure S3. Comparison of different allergens and their PNIF outcome. PNIF development compared between the different allergens and placebo. Both grass pollen and house dust mite (HDM) elicited significantly greater PNIF% reductions at each associated time of measurement than placebo (p < 0.001) and birch pollen (p < 0.01). PNIF% is displayed as medians and boxplots. Outliers are presented as degree sign, extreme outliers as asterisk. [file 13601_2017_169_MOESM7_ESM.pdf]
