# Supplementary material for: Peak nasal inspiratory flow as outcome for provocation studies in allergen exposure chambers: a GA2LEN study
Source: Clin Transl Allergy. 2017 Sep 17;7:33. doi: 10.1186/s13601-017-0169-4 (PMC5604509; doi:10.1186/s13601-017-0169-4)
Supplement: Supplementary file 6 — Additional file 6: Appendix S1. Comparison between the different allergens. [file 13601_2017_169_MOESM6_ESM.docx]

**Appendix S1 - Comparison between the different allergens**

To compare the different allergens with each other and placebo all active challenges of each allergen were pooled and medians of their PNIF% values were calculated. 203 challenges were conducted, in detail 56 challenges with grass pollen, 72 challenges with birch pollen, 24 challenges with HDM and altogether 51 placebo challenges (22 for grass, 22 for birch (11 of these runs were used for both grass and birch because the test subject was sensitized to both allergens) and 18 for HDM runs). Significant differences between grass pollen and placebo and HDM and placebo were found as suspected from results analyzed before. Moreover, both grass pollen and HDM had significant lower PNIF% values at each point of measurement during exposure than birch pollen when using all birch challenges (Additional file 7: *Fig S3*). If only those birch challenges were included where patients reached a TNSS greater 2 points on at least two symptom check cards as described before, no significant difference between grass pollen and birch pollen could be found, but still between HDM and birch pollen. Additionally, only when looking at the selected birch data a significant difference between birch pollen and placebo was detected for PNIF% values after 120 min of exposure. Almost every allergen and amount of allergen, respectively, reached a plateau after 60 min of exposure. However, active challenges with 8000 grains/m³ of grass pollen and 16000 grains/m³ of birch pollen even improved at the last point of measurement during exposure after 120 min, though still reaching lower values than their respective allergen challenges with fewer allergen concentration.

**Additional Figures**

**Figure S1** – **Title:** Reduction of PNIF during exposure with birch pollen in the GA²LEN chamber.

**Legend:** PNIF development during exposure with Betula pendula. Every challenge to birch pollen got pooled into one active group and only those runs were included where a TNSS greater than 2 points was reported on at least two symptom check cards throughout the whole challenge. A hash (#) marks a reduction compared to baseline p < .001, a dagger (†) a reduction compared to baseline p < .01, a double dagger (‡) a reduction compared to baseline p < .05. Outliers are presented as degree sign (°), extreme outliers as asterisk (*). PNIF% in the active group differed significantly (p < .05) from the placebo group at 60, 90 and 120 min. PNIF% is displayed as medians and boxplots.

**Figure S2** – **Legend:** Example of individual PNIF development (in L/min) for every subject when exposed to house dust mite (A – placebo, B – 250 µg/m³).

**Figure S3** – **Title:** Comparison of different allergens and their PNIF outcome.

**Legend:** PNIF development compared between the different allergens and placebo. Both grass pollen and house dust mite (HDM) elicited significantly greater PNIF% reductions at each associated time of measurement than placebo (p < .001) and birch pollen (p < .01). PNIF% is displayed as medians and boxplots. Outliers are presented as degree sign (°), extreme outliers as asterisk (*).
